# Association between *Schistosoma mansoni* infection and access to improved water and sanitation facilities in Mwea, Kirinyaga County, Kenya

**DOI:** 10.1186/s12879-019-4105-1

**Published:** 2019-06-07

**Authors:** Paul M. Gichuki, Stella Kepha, Damaris Mulewa, Janet Masaku, Celestine Kwoba, Gabriel Mbugua, Humphrey D. Mazigo, Charles Mwandawiro

**Affiliations:** 1 Eastern and Southern Africa Center for International Parasite Control (ESACIPAC), Kenya Medical Research Institute (KEMRI), P.O BOX 54840-00200, Nairobi, Kenya; 2 School of Health Sciences, Meru University of Science and Technology, P.O BOX 972-60200, Meru, Kenya; 3 London School of Tropical Medicine and Hygiene, Keppel St, Bloomsbury, London, WCIE 7HT UK; 4 School of Public Health, Pwani University, P.O BOX 195-80108, Mombasa, Kenya; 5 Vectorborne diseases Control Unit, Ministry of Health, P.o box 86-10303, Wanguru, Kenya; 6 Department of Medical Parasitology, School of Medicine, Catholic University of Health and Allied Sciences, P.O. Box 1464, Mwanza, Tanzania

**Keywords:** *Schistosoma mansoni*, Water, Sanitation, Improved, Unimproved, Kenya

## Abstract

**Background:**

Schistosomiasis remains a public health problem in Central Kenya despite concerted control efforts. Access to improved water and sanitation has been emphasized as important control measures. Few studies have assessed the association between access to improved water sources and sanitation facilities with *Schistosoma mansoni* infection in different environmental settings. This study assessed the association between *S. mansoni* infection and household access to improved water sources and sanitation facilities in Mwea, Kirinyaga County, Kenya.

**Methods:**

A cross sectional study was conducted between the months of August and October 2017. A total of 905 household heads from seven villages were interviewed and their stool samples screened for *S. mansoni* using the Kato Katz technique. Comparisons of demographic factors by *S. mansoni* infection were tested for significance using the chi-square test (χ2) or the Fisher exact test for categorical variables. Variables associated with *S. mansoni* infection were analyzed using univariable analysis and the strength of the association measured as odds ratio (OR) using mixed effects logistic regression at 95% CI, with values considered significant at *p* < 0.05.

**Results:**

The overall prevalence of *S. mansoni* was, 23.1% (95% CI: 20.5–26.0%), with majority of the infections being of light intensity. Rurumi village had the highest prevalence at 33.3%, with Kirogo village having the least prevalence at 7.0%. Majority (84.1%) of the households lacked access to improved water sources but had access to improved sanitation facilities (75%). Households with access to piped water had the lowest *S. mansoni* infections. However, there was no significant association between *S. mansoni* infections with either the main source of water in the household (Odds Ratio (OR) =0.782 (95% CI: 0.497–1.229) *p* = 0.285 or sanitation facilities (OR = 1.018 (95% CI: 0.705–1.469) *p* = 0.926.

**Conclusion:**

Our study suggests that *S. mansoni* is still a public health problem among all age groups in Mwea irrigation scheme, Kirinyaga County, Central Kenya. Majority of the households lacks access to improved water sources but have access to improved sanitation facilities. This study recommends initiatives to ensure adequate provision of improved water sources, and the inclusion of the adult community in preventive chemotherapy programs.

**Electronic supplementary material:**

The online version of this article (10.1186/s12879-019-4105-1) contains supplementary material, which is available to authorized users.

## Background

Schistosomiasis is a parasitic disease caused by a trematode worm of the genius *Schistosoma* [1]. There are two types of schistosomiasis, intestinal and urinary. Intestinal schistosomiasis is caused by *Schistosoma mansoni* and *Schistosoma japonicum* where parasite eggs are released in faeces while urinary schistosomiasis is caused by *Schistosoma haematobium*, and parasite eggs are released in the urine [1]. *Schistosoma mansoni*, transmitted by Biomphalaria snails and *Schistosoma haematobium,* transmitted by Bulinus snails are the most prevalent Schistosoma species [2]. According to the Global burden of disease report of 2013, more than 290 million people worldwide are estimated to be infected with schistosomiasis, about 600–780 million are at risk of infection, with morbidity due to these infections resulting to an estimated 2.8 million disability adjusted life years (DALYs) [2]. Schistosomiasis is endemic in more than 78 countries, with more than 90% of the infections occurring in sub- Saharan Africa [3].

In Kenya, approximately six million people have schistosomiasis and an additional fifteen million are at risk of infection [4]. Two species are predominant in Kenya, *Schistosoma haematobium* and*, Schistosoma mansoni* [5]. Recent findings reported prevalence of 2.1% for *Schistosoma mansoni*, and 14.8% for *Schistosoma haematobium* among school going children [6]. The distribution of Schistosomiasis in Kenya is such that *Schistosoma haematobium* is found mainly around the coast regions, some parts of Lake Victoria and Kano plains in Western Kenya [7], while *Schistosoma mansoni* occurs mainly in the Western parts of the country [8], and some parts of Central Kenya [9].

A school based schistosomiasis and soil transmitted helminths control programme was initiated in the year 2004 through collaboration between Kenya Medical Research Institute (KEMRI) and Japan International Corporation Agency (JICA). The programme entailed mass drug administration (MDA) of preventive chemotherapy to all school age children in Mwea, Kirinyaga County [10]. The preventive chemotherapy included a single dose of 40 mg/kg of Praziquantel administered using the tablet dose pole to determine the number of tablets to be taken by a child, and Albendazole as a single dose of 400 mg [11]. The programme was implemented until the year 2008, after which MDA was taken over by the Kenya National School based deworming programme.

Schistosomiasis contributes significantly to lower social economic conditions in areas where it is endemic and causes a great deal of disability thus reducing the work performance among the infected individuals. However, mortality associated with these infections is low [12]. Schistosomiasis infections have been shown to increase the susceptibility to or severity of co-infecting pathogens [1], and as a result the disease has been targeted for control and eventual elimination by the World Health Organization (WHO) [13].

Like many other endemic countries, the control of schistosomiasis is through mass drug administration (MDA) using the drug of choice Praziquantel [13]. Although chemotherapy is cost-effective [14] and reduces schistosome infections in human hosts [15], it has a limitation in that it does not kill immature worms [16] and has low impact on transmission [17]. This intervention is often delivered through school based deworming programme (SBDP) and offers many benefits to the treated children [18].

Recently, there has been global advocacy geared towards schistosomiasis transmission interruption, with a call in the year 2012 by the World Health Assembly (WHA) resolution 65.21, on countries to intensify control and initiate elimination campaigns [19]. Water, sanitation and hygiene education (WASH) have been emphasized as a component of an integrated control and elimination strategy in the WHA resolution on the bases that they should reduce schistosomiasis transmission by reducing human water contact [20]. WASH has been acknowledged in WHO prevention and control guidelines, which advices the inclusion of the same in helminth control programs [21].

The provision of access to safe drinking water, hygiene and sanitation, which has also been classified as the “forgotten foundations of health” [22, 23], though essential in the control of schistosomiasis, is inadequate in large parts of low and middle-income countries where schistosomiasis is endemic [1, 24]. WASH interventions have the potential to reduce the environmental exposure to infected schistosome eggs and larvae and thus reduce transmission of the disease ensuring a long term improvement in people’s wellbeing [25, 26].

Even though the important role of WASH has been recognized and advocated for in the World Health Assembly (WHA) resolutions on schistosomiasis [27], WASH has not been incorporated in disease specific control programs. For this to happen, there is need for data on the levels of community access to clean water, sanitation and hygiene and how each aspect of WASH associates with schistosomiasis infection in different endemic settings. This will help us understand which specific WASH intervention is most effective in reducing exposure to infection in what environmental setting. Several studies have reported on the effects of WASH on NTDs especially STHs [28–33], however, little evidence exists to inform policy decisions about the importance of including WASH as part of schistosomiasis control. Therefore, the aim of this study was to describe the association between *S. mansoni* infection and household access to improved water sources and sanitation facilities in Kirinyaga County, central Kenya.

## Methods

### Study area

The present study was conducted in two different Sub Counties of Kirinyaga County. The Sub Counties included Mwea East and West. Kirinyaga County lies between 1158 M and 5380 M above sea level in the South and at the Peak of Mount Kenya respectively, with a mean annual rainfall ranging between 1200 and 1600 mm per year. Covering an area of 1478.1 km^2^, the County is located about 100kms north east of Nairobi. Figures from the 2009 census indicate that the County had an estimated total population of 528,054 persons, with an annual growth rate of 1.5%. The population was projected to be 593,379 in the year 2017 [34]. There were an estimated 154, 220 households in the county. The two Sub Counties are home to the giant Mwea irrigation scheme where several water canals crisscross the area supplying irrigation water to the farms and villages respectively. The main socio-economic activities include rice and horticultural farming. Generally, Mwea East and West Sub Counties are endemic for *S. mansoni* [9, 35, 36]. However, data on community access to improved water and sanitation is lacking.

### Study population and selection criteria

**Fig. 1:**
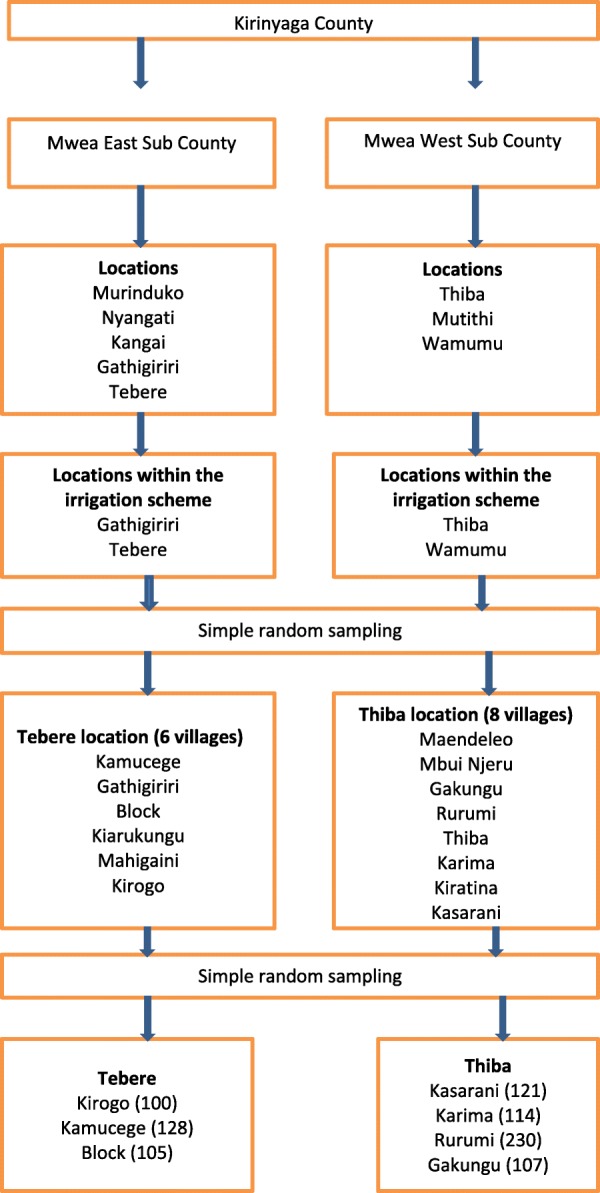
Flow diagram showing selection of study villages

**Fig. 2:**
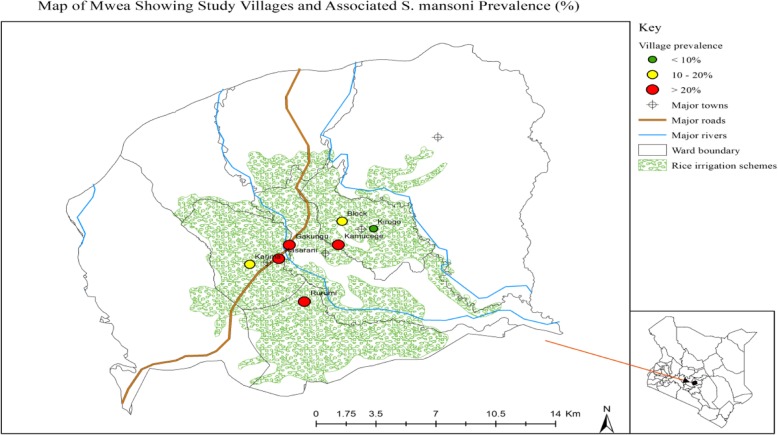
Map of Mwea, showing the study villages and associated *S. mansoni* prevalence

In Mwea West Sub County, there were two locations both of which were located within the irrigation scheme (Thiba and Mutithi); while in Mwea East there were a total of five locations. These included Gathigiriri, Tebere, Murinduko, Nyangati and Kangai. Tebere and Gathigiriri locations were located within the Mwea irrigation scheme. Tebere and Gathigiriri locations in Mwea East and Thiba and Mutithi in Mwea West were included in the study since they were located within the irrigation scheme. Tebere and Thiba locations from Mwea East and West respectively were then sampled randomly and included in the study. Tebere location had a total of six villages which included Kamucege, Gathigiriri, Block, Kiarukungu, Mahigaini and Kirogo, while Thiba had eight villages including Mbui Njeru, Maendeleo, Gakungu, Rurumi, Thiba, Karima, Kiratina and Kasarani. Four and three villages from Thiba and Tebere locations respectively were selected and included in the study (Fig. 1). The study villages are shown in Fig. 2.

### Study design and sample size determination

This was a cross sectional study carried out between the months of August and October 2017. The study used prevalence of 26% for *Schistosoma mansoni* derived from a recent study in the area [35], level of significance and an error margin of 0.05, and standard deviation at 95% CI (1.96) to calculate the sample size [37]. A sample size of 432 per sub County was calculated, and multiplied by two since the study was covering two sub Counties to give a minimum sample size of 864 which was then adjusted to 905 to cater for refusals and incomplete data.

Lists of all households per each village were obtained from the village Community Health Workers (CHW). Using probability proportional to size sampling and the total number of households per village, each village was allocated a minimum number of households to be sampled from the total study sample size. In each village, the first household to be sampled was identified by the study team and systematic random sampling used to locate every third household during the survey. In each household sampled, the head or a representative of the sampled household was interviewed. The study team held meetings with the leadership at the County and Sub County level, the local administration and village members in all the seven study villages prior to the start of data collection to explain to them the purpose of the study and seek their consent. Each participating household head gave individual written consent before the survey.

### Questionnaire administration

A structured questionnaire developed in English and translated to the local language (kikuyu) was administered to the household heads in selected households. The questionnaire included sections on social demographic indicators such as age, sex, marital status, religion, education, occupation, and the assessment of water and sanitation. The section on water and sanitation was developed based on the definitions by the joint monitoring programme (JMP), of the World Health Organization (WHO) and the United Nations Children Fund (UNICEF), for water and sanitation [38]. Improved sanitation facilities was assessed in households that had flush toilet, a piped sewer system, a septic tank, flush/ pour to a pit latrine, ventilated improved pit latrine (VIP), pit latrine with slab and a composting toilet. Unimproved toilet facilities were assessed in households that had flush pour to elsewhere, pit latrine without slab, bucket, hanging toilet/ latrine or no facility at all. Improved water source were assessed in households which had access to piped water into dwelling, piped water to yard/ plot, public tap or stand pipe, tube well or borehole, protected dug well, protected spring, bottled water and rain water, while Unimproved water source included unprotected spring, unprotected dug well, cart with small tank, tanker-truck and surface water. Surface water included rivers, dams, lakes, ponds, streams, canals and irrigation channels. Additional information in this section included time taken to the water source and whether the household head was satisfied with the source. Presence of sanitation facility and water source in the household, whether there were faeces on the toilet floor, and whether or not the toilet was in use was observed by physically visiting the facility. Information on toilet sharing was also collected (Additional file 1).

### Parasitological screening for *S. mansoni*

Household heads or their adult representatives were asked to provide a stool sample. A clean screw capped, well labeled plastic container with scoop was provided with instructions on how to collect a stool sample. Those who were not able to provide a stool sample were visited the next day until they got the sample. The samples were collected the same day or the following morning and taken to Kimbimbi Sub County hospital laboratories for analysis. Samples that could not be processed the same day were stored at 4 °C. In the laboratory, Kato Katz techniques was used in sample processing where each sample was prepared in duplicate smears of 41.7 mg [39]. A random sample of 10% of all the positive and negative slides read each day were randomly reexamined by a third experienced laboratory technologists to ensure quality work.

### Data analysis

Household data collected was entered and stored in an excel spreadsheet and counter checked for accuracy. Parasitology data was entered in the laboratory parasitology result forms and then entered in the excel spreadsheet. Data was analyzed using STATA version 14.0 (Stata Corporation, College Station, TX, USA). Presence of *S. mansoni* egg across the duplicate slides indicated infection while the arithmetic mean of eggs per gram (epg) of faeces across the duplicate slides expressed the intensity of the infection. The infection intensity was categorized according to the WHO classifications for *S. mansoni* (0) negative, (1–99) light, (100–399) moderate and (> 400) heavy [40]. The prevalence of *S. mansoni* was calculated by age group, gender and village of residence. Comparisons of demographic factors by *S. mansoni* infection were tested for significance using the chi-square test (χ2) or the Fisher exact test for categorical variables. Factors associated with *S. mansoni* infection were analyzed using univariable analysis and the strength of the association measured as odds ratio (OR) using mixed effects logistic regression at 95% CI, with values considered significant at *p* < 0.05.

### Ethical considerations

Prior to the implementation of the study, ethical approvals were sought from the Scientific and Ethical Review Unit (SERU) of the Kenya Medical Research Institute (KEMRI), number (KEMRI/SERU/ESACIPAC/007/3326) and Meru University of Science and Technology Institutional Research Ethics Review Committee (MIRERC), number (MIRERC/001/2017), and the Health Management Team of Kirinyaga County. All the household heads participating in the study signed a written informed consent which had been translated into the local dialect. Those who could not read or write were asked to have an independent person as a witness who ensured that the study was clearly explained to them and guided them to give a thumb print on the consent form.

## Results

### Demographic information of the study participants

Overall, data was collected from a total of 905 household heads who gave a written informed consent, 70.5% of whom were female. The age of the respondents ranged from 17 to 95 years. Respondents within the age bracket of 36 and 45 years were the majority 30.3%, followed by those in the age bracket of 26 and 35 years at 28.8%. Majority of the respondents were Christians (95.0%), married (83.5%) and farmers (63.6%). Those who had no formal education were 7.6%, most of who hailed from Karima village as shown in Table 1.

**Table 1:** Household demographic factors aggregated by villages

Characteristic	Karima N (%)	Rurumi N (%)	Kasarani N (%)	Kirogo N (%)	Kamucege N (%)	Block N (%)	Gakungu N (%)	Total
Sex								
Male	34 (29.8)	106 (46.1)	22 (18.1)	25 (25.0)	33 (25.8)	28 (26.7)	19 (17.8)	267 (29.5)
Female	80 (70.2)	124 (53.9)	99 (81.9)	75 (75.0)	95 (74.2)	77 (73.3)	88 (82.2)	638 (70.5)
Age (years)								
≤ 25	15 (13.2)	21 (9.1)	9 (7.4)	6 (6.0)	12 (9.4)	11 (10.5)	15 (14.0)	89 (9.8)
26–35	22 (19.3)	69 (30.0)	37 (30.6)	25 (25.0)	30 (23.4)	37 (35.2)	41 (38.8)	261 (28.8)
36–45	27 (23.7)	72 (31.3)	39 (32.2)	29 (29.0)	37 (28.9)	34 (32.4)	33 (30.9)	271 (30.0)
46–55	21 (18.4)	30 (13.0)	30 (24.8)	24 (24.0)	23 (18.0)	8 (7.6)	14 (13.1)	150 (16.6)
> 55	29 (25.4)	38 (16.6)	6 (5.0)	16 (16.0)	26 (20.3)	15 (14.3)	4 (3.7)	134 (14.8)
Marital status								
Married	97 (85.1)	179 (77.8)	105 (86.8)	89 (89.0)	109 (85.2)	83 (79.0)	94 (87.9)	756 (83.5)
Single/ Window(wer)/ Divorced	17 (14.9)	51 (22.2)	16 (13.2)	11 (11.0)	19 (14.8)	22 (21.0)	13 (12.1)	149 (16.5)
Religion								
Christian	106 (93)	220 (95.7)	116 (95.8)	95 (95.0)	117 (91.4)	100 (95.2)	106 (99.1)	860 (95.0)
Others	8 (7.0)	10 (4.3)	5 (4.2)	5 (5.0)	11 (8.6)	5 (4.8)	1 (0.9)	45 (5.0)
Education								
No formal education	20 (17.5)	12 (5.2)	5 (4.1)	7 (7.0)	16 (12.5)	10 (9.5)	2 (1.9)	72 (7.6)
Primary (Incomplete)	31 (27.2)	72 (31.3)	38 (31.4)	26 (26.0)	42 (32.8)	37 (35.2)	35 (32.7)	280 (20.9)
Primary (Complete)	41 (36.0)	107 (46.5)	45 (37.2)	48 (48.0)	47 (36.8)	38 (36.2)	41 (38.3)	370 (40.9)
Secondary Incomplete)	20 (17.5)	31 (13.5)	26 (21.5)	16 (16.0)	20 (15.6)	14 (13.3)	23 (21.5)	152 (16.8)
Secondary and post-secondary	2 (1.8)	8 (3.5)	7 (5.8)	3 (3.0)	3 (2.3)	6 (5.8)	6 (5.6)	31 (13.8)
Occupation								
Business	8 (7.0)	24 (10.4)	26 (21.5)	13 (13.0)	15 (11.7)	17 (16.2)	20 (18.7)	123 (13.6)
Casual labourers	9 (7.9)	47 (20.4)	18 (14.9)	10 (10.0)	19 (14.8)	20 (19.0)	23 (21.5)	146 (16.1)
Others	8 (7.0)	13 (5.7)	18 (14.9)	1 (1.0)	4 (3.2)	6 (5.8)	11 (10.3)	61 (6.7)
Farmer	89 (78.1)	146 (63.5)	59 (48.7)	76 (76.0)	90 (70.3)	62 (59)	53 (49.5)	575 (63.6)

N = 905 Karima N = 114, Rurumi N = 230, Kasarani N = 121, Kirogo N = 100, Kamucege N = 128, Block N = 105, Gakungu =107

### Household access to improved water and sanitation facilities

Majority of the households (84.1%) had access to unimproved water sources mainly from surface water sources such as canals, rivers and streams. Other sources of water included piped water to plot (5.7%), borehole (5.6%) and rainwater collection (4.0%). Kasarani village had the highest households with access to piped water (19.0%), while Kirogo village had the highest access to borehole water at 49.0%. Majority of the households had improved sanitation facilities (74.9%), where the most common type was the VIP/simple latrine with concrete floor slab. Pit latrine without floor slab was the most common unimproved type at (21.9%). Rurumi village had the highest percentage (4.7%) of households without a sanitation facility (Table 2).

**Table 2:** Household access to water sources and sanitation facilities aggregated by villages (*N* = 905)

Characteristic	Karima N (%)	Rurumi N (%)	Kasarani N (%)	Kirogo N (%)	Kamucege N (%)	Block N (%)	Gakungu N (%)	Total
Source of water								
Piped water	0 (0)	7 (3.0)	19 (15.7)	9 (9.0)	0 (0)	0 (0)	13 (12.1)	48 (5.3)
Borehole	0 (0)	0 (0)	0 (0)	49 (49.0)	0 (0)	0 (0)	0(0)	49 (5.4)
Protected dug well	0 (0)	0(0)	0 (0)	0 (0)	0 (0)	10(9.5)	6 (4.7)	6 (0.7)
Rainwater collection	2 (1.8)	21 (9.1)	2 (1.7)	1 (1.0)	0 (0)	2 (1.9)	4 (3.7)	32 (3.5)
Other sources	0 (0)	0 (0)	5 (4.1)	0 (0)	0 (0)	0 (0)	0(0)	5 (0.6)
Surface water/ unprotected dug well	112(98.2)	202 (87.2)	95 (78.5)	41 (41.0)	128 (100)	103 (98.1)	84 (78.5)	765 (84.5)
Time taken to fetch water								
On premises	0 (0)	59 (25.7)	93 (76.9)	49 (49.0)	19 (14.8)	23 (21.9)	54 (50.5)	297 (32.8)
Less than thirty minutes	111 (97.4)	147 (63.9)	28 (23.1)	51 (51.0)	109 (85.2)	82 (78.1)	50 (46.7)	578 (63.9)
More than thirty minutes	3 (2.6)	24 (10.4)	0 (0)	0 (0)	0 (0)	0 (0)	3 (2.8)	30 (3.3)
Sanitation facility								
VIP/Simple latrine with floor slab	98 (86.0)	149 (64.8)	104 (85.9)	91 (91.0)	87 (68.0)	60 (57.1)	89 (83.2)	678 (74.8)
Pit latrine without floor slab	16 (14)	62 (27.0)	12 (9.9)	7 (7.0)	38 (29.7)	45 (42.9)	18 (16.8)	198 (21.9)
Other	0 (0)	8 (3.5)	5 (4.2)	2 (2.0)	0 (0)	0 (0)	0 (0)	15 (1.7
No facility	0 (0)	11 (4.7)	0 (0)	0 (0)	3 (2.3)	0 (0)	0 (0)	14 (1.6)
Reported sanitation access								
Shared	25 (78.1)	45 (20.5)	30 (24.8)	25 (25.0)	33 (25.8)	18 (17.1)	18 (16.8)	194 (21.7)
Not shared	89 (21.9)	174 (79.5)	91 (75.2)	75 (75.0)	95 (74.2)	87 (82.9)	89 (83.2)	700 (78.3)
Toilet in use								
Yes	114 (100)	226 (98.3)	120 (99.2)	100 (100)	0 (0)	103 (98.1)	107 (100)	898 (99.2)
No	0 (0)	4 (1.7)	1 (0.8)	0 (0)	0 (0)	2 (1.9)	0 (0)	7 (0.8)
Visible faeces on the edges								
Yes	17 (14.9)	52 (27.4)	10 (8.3)	21 (21.0)	37 (28.9)	31 (29.5)	20 (18.7)	188 (21.0)
No	97 (85.1)	167 (72.6)	111 (91.7)	79 (79.0)	91 (71.1)	74 (70.5)	87 (81.3)	706 (79.0)
Household has child (ren) under 3 years								
Yes	17 (14.9)	74 (32.2)	37 (30.6)	28 (28.0)	26 (20.3)	19 (18.1)	47 (43.9)	248 (27.4)
No	97 (85.1)	156 (67.8)	84 (69.4)	72 (72.0)	102(79.7)	86 (81.9)	60 (56.1)	657 (72.6)
Disposal of feaces for under 3 years								
Child used toilet/ latrine	1 (6.0)	15 (20.3)	12 (32.4)	8 (28.6)	13 (50)	13 (68.4)	16 (30.1)	78(31.4)
Other	16 (94.0)	55 (74.3)	24 (64.9)	17 (60.7)	12 (46.2)	0 (0)	30 (63.8)	154 (62.1)
Put/ rinsed in toilet/ latrine	0 (0)	4 (5.4)	1 (0.7)	3 (10.7)	1 (3.8)	6 (31.6)	1 (6.1)	16 (6.5)

N = 905 Karima N = 114, Rurumi N = 230, Kasarani N = 121, Kirogo N = 100, Kamucege N = 128, Block N = 105, Gakungu =107

### Prevalence and intensity of *S. mansoni* infections

The overall prevalence of *S. mansoni* was 23.1% (95% CI: 20.5–26.0%). The highest prevalence was reported in Rurumi village (31.3%), followed by Kasarani, Gakungu and Kamucege villages (28.1, 25.2 and 23%) respectively. Kirogo village had the least infection. Compared to Gakungu, residents of Kirogo village had about 78% lower odds of having *S. mansoni* infection (OR) 0.223(95% CI 0.092–0.540), *p* < 0.001). The burdens of *S. mansoni* infections were not significantly different in all the other villages. The number of *S. mansoni* eggs observed in the specimens ranged from 12 to 8148 eggs per gram (epg) of faeces. Majority of the *S. mansoni* infections (75%) were of light intensity (Table 3).

**Table 3:** Prevalence and Intensity of *Schistosoma mansoni* infections by villages

	Karima N (%)	Rurumi N (%)	Kasarani N (%)	Kirogo N (%)	Kamucege N (%)	Block N (%)	Gakungu N (%)	Total
*S. mansoni*								
Positive	21	72	34	7	29	19	27	209
Negative	93	158	87	93	99	86	80	696
Prevalence	18.4	31.3	28.1	7	22.7	18.1	25.2	23.1
OR (95% CI)	0.669(0.351–1.274)	1.350(0.805–2.266)	1.158(0.642–2.088)	0.223(0.092–0.540)	0.868(0.476–1.584)	0.655(0.338–1.268)	REF	
*P*-value	0.220	0.255	0.626	< 0.001	0.644	0.207		
Intensity								
Light (1–99 epg)	20	46	19	6	24	17	24	156
Moderate (100–399 epg)	1	19	13	1	4	1	2	41
Heavy (≥400 epg)	0	7	2	0	1	1	1	12

N = 905 Karima N = 114, Rurumi N = 230, Kasarani N = 121, Kirogo N = 100, Kamucege N = 128, Block N = 105, Gakungu =107

### Association between *S. mansoni* infection and demographic variables

Table 4 summarizes the results for the association between *S. mansoni* infection and household demographic variables. Generally, male participants had the highest infection than female participants, though not statistically significant (26 and 22% respectively, *p* = 0.237). Households whose heads were farmers had the highest infection (24%) as compared to those whose occupation was business (19.8%) although not statistically significant *p* = 0.600. There were no statistical significant associations between *S. mansoni* infections and participant’s other social demographic attributes including education and marital status.

**Table 4:** Association between *S. mansoni* infection and demographic variables

Variables	*S. mansoni*		OR (95% CI)	*P*-value
	Positive	Negative		
Sex				
Female	141(22.1)	497(77.9)	REF	
Male	68(25.5)	199(74.5)	1.204(0.863–1.680)	0.273
Total	209	696		
Age (years)				
> 55	36(26.1)	102(73.9)	REF	
46–55	36(24.2)	113(75.8)	0.903(0.529–1.540)	0.707
36–45	53(19.3)	221(80.7)	0.679(0.419–1.102)	0.116
26–35	69(25.7)	199(74.3)	0.982(0.615–1.569)	0.941
≤ 25	15(19.7)	61(80.3)	0.697(0.353–1.376)	0.297
Total	209	696		
Marital status				
Single/Widow(er)/Divorced	66(22.5)	227(77.5)	REF	
Married	143(23.4)	469(76.6)	1.049(0.752–1.462)	0.779
Total	209	696		
Religion				
Others	6(13.6)	38(86.4)	REF	
Christian	203(23.6)	658(76.4)	1.954(0.814–4.688)	0.127
Total	209	696		
Education				
Secondary & Post-secondary	8(25.8)	23(74.2)	REF	
Secondary (Incomplete)	28(18.7)	122(81.3)	0.660(0.267–1.628)	0.365
Primary (Complete)	82(22.0)	291(78.0)	0.810(0.349–1.878)	0.623
Primary (Incomplete)	78(27.9)	202(72.1)	1.110(0.476–2.587)	0.809
No formal education	13(18.3)	58(81.7)	0.644(0.236–1.759)	0.389
Total	209	696		
Occupation				
Farmer	50(8.7)	522(91.3)	REF	
Business person	64(52.0)	59(48.0)	1.248(0.845–1.843)	0.265
Casual labourer	68(43.3)	89(56.7)	0.879(0.616–1.255)	0.477
Others	27(50.9)	26(49.1)	1.195(0.680–2.098)	0.535
Total	209	696		
Village				
Gakungu	27(25.2)	80(74.8)	REF	
Karima	21(18.4)	93(81.6)	0.669(0.351–1.274)	0.220
Rurumi	72(31.3)	158(68.7)	1.350(0.805–2.266)	0.255
Kasarani	34(28.1)	87(71.9)	1.158(0.642–2.088)	0.626
Kirogo	7(7.0)	93(93.0)	0.223(0.092–0.540)	< 0.001
Kamucege	29(22.7)	99(77.3)	0.868(0.476–1.584)	0.644
Block	19(18.1)	86(81.9)	0.655(0.338–1.268)	0.207
Total	209	696		

N = 905

**Fig. 3:**
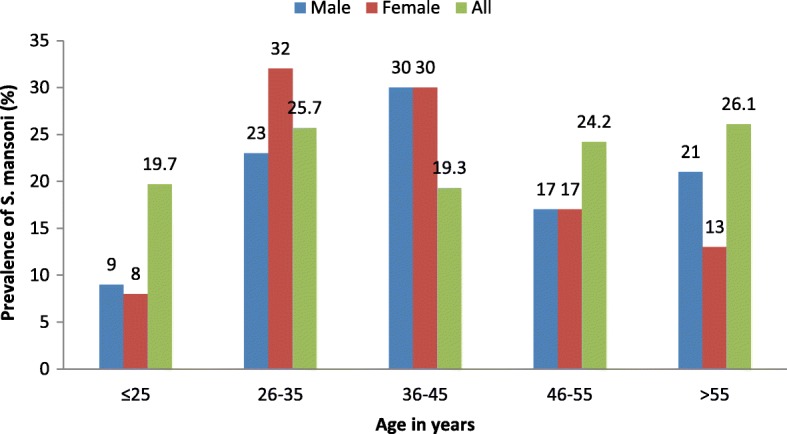
Distribution of *S. mansoni* infections by age and gender

Infection with *S. mansoni* was highest among participants of more than 55 years of age (26.1%), followed by the age group 26 to 35 years (25.7%). The lowest prevalence of *S. mansoni* infections was recorded among study participants of age group 36–45 years (19.3%). In the age group ≤25 years and > 55 years, male participants had higher prevalence compared to female participants. In the 26 to 35 years’ age group men had lower prevalence of *S. mansoni* infections compared to women (23 and 32% respectively) (Fig. 3).

### Association between *S. mansoni* infections, water and sanitation variables

A total of 138 (15.2%) households had access to improved water sources. Out of these households, 27 (19.6%) household heads were infected with *S. mansoni.* Among the various sources of water, participants from households utilizing piped water had the lowest proportion of infections with *S. mansoni* (13%). Prevalence of *S. mansoni* infection were 14, 24, 31 and 50% among the participants who reported that their main source of drinking water was borehole, unprotected dug well/surface water, rainwater collection and protected dug well respectively*.* Nonetheless, there was no significant association between the main sources of water in households and infection with *S. mansoni*, (OR = 0.782 (95% CI: 0.497–1.229) *p* = 0.285. Being satisfied with the water supply was a significant predictor of *S. mansoni* infection (*p* < 0.004). Those who reported that they were satisfied with the water supply had 38% reduction in odds of being found to be infected with *S. mansoni* when assessed against their counterparts whose opinion was on the contrary (OR 0.623 (95% CI 0.452–0.859). Other attributes of water consumed in the study households that were assessed in the study, including treating of water before use, time taken to fetch water and main person who fetches water for the household, did not show significant association with the infection.

A total of 678 (74.9%) of the study households had access to an improved sanitation facility. Out of these, a total of 148 (21.8%) household heads were infected with *S.mansoni.* Households with unimproved sanitation facilities were 227 (25.1%), out of which 61 (26.9%) household heads were infected with *S. mansoni*. However, there was no significant association between the type of sanitation facility (improved / unimproved) and *S. mansoni* infection (OR = 1.018 (95% CI: 0.705–1.469 *p* = 0.926(Table 5).

**Table 5:** – Association between *S. mansoni* infections, water and sanitation variables

Variables	*S. mansoni*		OR (95% CI)	*P*-value
	Positive	Negative		
Source of water in the household				
Unprotected dug well/Surface water	180(23.5)	585(76.5)	REF	
Piped	6(12.5)	42(87.5)	0.464(0.194–1.110)	0.078
Borehole	7(14.3)	42(85.7)	0.542(0.239–1.227)	0.136
Protected dug well	3(50.0)	3(50.0)	3.250(0.650–16.243)	0.149
Rainwater collection	10(31.3)	22(68.8)	1.477(0.687–3.178)	0.315
Other	3(60.0)	2(40.0)	4.875(0.808–29.403)	0.056
Total	209	696		
Time taken to fetch water from the source				
More than 30 min	16(26.2)	45(73.8)	REF	
On premises	64(22.4)	222(77.6)	1.112(0.463–2.672)	0.812
Less than 30 min	129(23.1)	429(76.9)	1.160(0.494–2.725)	0.733
Total	209	696		
Satisfied with the water supply (source)				
Not satisfied	136(26.7)	374(73.3)	REF	
Satisfied	73(18.5)	322 (81.5)	0.623 (0.452–0.859)	0.004
Total	209	696		
Drinking water classification				
Un improved	182(23.7)	585(76.3)	REF	
Improved	27(19.6)	111(80.4)	0.782(0.497–1.229)	0.285
Total	209	696		
Toilet/Sanitation facility type				
No facility	1(7.1)	13(92.9)	REF	
VIP/simple pit latrine with floor/slab	148(21.8)	530(78.2)	1.755(0.210–14.693)	0.507
Pit latrine without floor/slab	46(23.2)	152(76.8)	1.852(0.217–15.786)	0.999
Other	14(93.3)	1(6.7)	2.400(0.264–21.787)	0.661
Total	209	696		
Toilet shared with other households				
Not shared	154(22.2)	540(77.8)	REF	
Shared	54(27.4)	143(72.6)	1.149(0.791–1.668)	0.466
Total	208	683		
Toilet in use				
No	6(66.7)	3(33.3)	REF	
Yes	202 (22.9)	680(77.1)	0.400(0.089–1.802)	0.207
Total	208	683		
Presence of faeces on the floor				
No	163(23.2)	541(76.8)	REF	
Yes	45(24.1)	142(75.9)	1.069(0.730–1.564)	0.732
Total	208	683		
Household has child(ren) under 3 years				
No	161(22.4)	558(77.6)	REF	
Yes	48(25.4)	138(74.6)	1.195(0.820–1.742)	0.353
Total	209	696		
Disposal of faeces for children < 3 years				
Put/rinsed into toilet or latrine	42(25.0)	124(75.0)	REF	
Child used toilet/latrine	3(33.3)	6(66.7)	1.500(0.359–6.270)	0.695
Other	3(27.3)	8(72.7)	1.125(0.285–4.441)	0.999
Total	48	138		
Sanitation facility type				
Un-improved	61(26.9)	166(73.1)	REF	
Improved	148(21.8)	530(78.2)	1.018(0.705–1.469)	0.926
Total	209	696		

N = 905 + Disposal of faeces for children < 3 years, *n* = 185

## Discussion

The control intervention for most NTDs including Schistosomiasis is largely focused on preventive chemotherapy which is implemented through school based programs. However, there has been growing interest to include the adult communities and WASH interventions in the preventive chemotherapy programs. The current study has demonstrated that *S. mansoni* is a public health problem among the population living in Mwea, Kirinyaga County. The *S. mansoni* prevalence recorded in this study 23.1% (95% CI: 20.5–26.0%) places the study area under the WHO classification of moderate- risk communities [41]. Majority of the infections reported in this study were of light intensity which is consistent with findings from Western Kenya [42] and also supports previous findings that most individuals in endemic areas excrete low number of eggs [43].

In areas of moderate schistosomiasis risk, WHO recommends preventive chemotherapy as a strategy for morbidity control that will help lessen the occurrence and severity of consequences of infection for risk groups including irrigation workers, fishermen and women of child bearing age [44]. This result therefore adds to the growing concern that the adult community needs to be included in schistosomiasis control programmes since they may provide an avenue for continued re- infection of the school going children who are usually targeted for treatment in the school-based deworming programmes. Similar observations have been made in previous studies [6, 45, 46].

In this study, male participants were more at risk of infection with *S. mansoni* than female participants which is consistent with previous findings [47, 48]. This could be explained by the fact that, in most communities, male are more exposed to frequent water contact partly due to their economic activities like farming. This has been observed in other studies [49–52]. Participants who were advanced in age (> 55 years) were the most affected with *S. mansoni at* 26.1%. *Schistosoma mansoni* is a chronic infection which means that a person can live with the infections for a long time without seeking treatment. Similar findings have been reported elsewhere [9].

Majority of the households in the study had inadequate access to improved sources of water; however, there was no significant association between household water sources and *S. mansoni* infections. The national coverage for access to improved water in Kenyan rural areas stands at 59% [53], while the global coverage as at 1990 was 76% [38]. Previous studies have observed that, most countries where schistosomiasis is endemic have inadequate access to clean water sources [1, 24]. Surface water from open water bodies such as rivers, streams and irrigation channels, was the most common source of water for majority of the study participants. This has previously been observed elsewhere by Tchuem Tchuenté et al. who noted that natural water bodies many of which are infested with snails and infective schistosome cercariae are common sources for domestic water in most schistosomiasis endemic areas [54].

Usually people become infected with schistosomiasis when they come into contact with infested water and cercariae penetrate the skin. Schistosome eggs are then excreted in human faeces or urine and when they get into water bodies, miracidia which are released from the eggs in turn infects the snails, which release cercariae which penetrates human skin [1].

Households whose heads had access to piped water had the lowest infection with *S. mansoni* among those with improved water sources at 13%. Infection was highest among those who utilized improved water from protected dug well at 50%. The results further show that there was no significant association between household water sources and *S. mansoni* infection. Previous studies have reported strong association between access to improved water sources; with significantly less infection with *S. mansoni* [45, 55–57]. Another study linked lack of access to clean water sources to 47% population attribute ‘able fraction (PAF) of schistosomiasis [58]. Improved water sources may not contain cercariae, but even so, its provision may not prevent all water contact with infested water. Therefore, when looking at the relationship between water and *S. mansoni* infection, there is need to also consider the environmental set up since areas under flood irrigation e.g. rice irrigation areas may be different from other areas.

Mwea is a rice irrigation scheme where people live in clustered villages which are surrounded by vast rice fields, and water from the rice drainage flows freely in to canals and streams which cuts across the villages, and thus even when a household has access to improved water sources, there could be high chances of them coming into contact with water from unimproved sources. Previous studies have alluded to this. Groups like irrigation workers and canal cleaners have suffered high exposure to infested water in the People’s Republic of China [59, 60]. Another study in Brazil found out that people who crossed streams were at significantly higher risk of *S. mansoni* infection [61].

In regards to household access to sanitation facilities, the present study did not find an association between the type of sanitation facility (Improved or unimproved) with *S. mansoni* infections. This is in contrast to other findings which have reported significant lower odds of *S. mansoni* infection among people with access to adequate sanitation [57, 62]. In order to sustain transmission, a schistosome egg must enter freshwater to infect snail which will then release cercariae which infects people who come into contact with water [1]. For sanitation to be effective in controlling schistosomiasis, it should be able to contain fecal matter and urine hence preventing the schistosome eggs from hatching and thus ensuring that there are no miracidia to infect the snails. Studies have demonstrated that schistosome reproduces exponentially within the host snail, and therefore even small numbers of eggs entering freshwater may give rise to high risk of infection to people coming into contact with the water [63]. Studies have shown that even when high sanitation coverage levels are achieved, their use may still remain low [64, 65], due to various behavioral factors [66]. Open water bodies have been shown to be particularly attractive sites for open defecation [67]. The study area being a rice irrigation scheme, where sanitation facilities are not provided for in the farms, there are possibilities of open defecation when people are working in the farms. This has been demonstrated by Chimbari et al. [68].

We acknowledge that our sample selection process could be a potential source of bias since we used the households as unit of randomization and individuals as the units of study. We however used an effect of design of two for the sample size calculation which gave us a larger sample so as to minimize this bias. Secondly, we did not factor in data on possible behavioural factors such as open defecation which could also play a role in schistosomiasis transmission. Future studies may consider incorporating this aspect. Also, the study analysed single stool sample using the Kato Katz technique which might have missed light infections because of its poor sensitivity and day to day fluctuations in egg excretion [69]. Future population studies may enhance this by collecting stool samples for at least two consecutive days.

## Conclusion

In conclusion, this study reaffirms that *S. mansoni* is still a public health problem among communities living in Kirinyaga County. The study also shows that individuals of all age groups are infected with *S. mansoni* and thus supports the call for inclusion of the adult community in targeted mass drug administration. There was inadequate access to improved water sources in the study area but adequate access to improved sanitation facilities. However, there was no significant association between *S. mansoni* infection with either access to improved water sources or sanitation facilities. The findings of this study add to the existing knowledge on the association between specific WASH components and *Schistosoma mansoni* infections in areas where rice irrigation farming is practiced. The study recommends further investigations on the association of schistosomiasis infections and hygiene in similar set ups.

## Additional file

Additional file 1: Household questionnaire. (DOCX 26 kb)

## Data Availability

Please contact the corresponding author for data requests.

## Abbreviations

CHWs: Community Health Workers
CI: Confidence interval
DALYs: Disability Adjusted Life Years
Epg: Eggs per gram of faeces
ESACIPAC: Eastern & Southern Africa Center of International Parasite Control
IQR: Interquartile range
JICA: Japan International Corporation Agency
JMP: Joint Monitoring Programme
KDHS: Kenya demographic and health surveys
KEMRI: Kenya medical research institute
MDA: Mass drug administration
MDG: Millennium development goals
MIRERC: Meru Univeristy of Science and Technology Institutional Research Ethics Research Committee
NTDs: Neglected tropical diseases
OR: Odds ratio
SBDP: School based deworming program
SERU: Scientific and ethical review unit
UNICEF: United Nations children’s fund
VIP latrines: Ventilated improved latrines
WASH: Water sanitation and hygiene
WHO: World health organization
