# Supplementary material for: Association between Schistosoma mansoni infection and access to improved water and sanitation facilities in Mwea, Kirinyaga County, Kenya
Source: BMC Infect Dis. 2019 Jun 7;19:503. doi: 10.1186/s12879-019-4105-1 (PMC6556037; doi:10.1186/s12879-019-4105-1)
Supplement: Supplementary file 1 — Household questionnaire. (DOCX 26 kb) [file 12879_2019_4105_MOESM1_ESM.docx]

**Household Questionnaire**

**Location ………………………………………………………………………………...**

**Village…………………………………………………………………………………..**

**Name of interviewer……………………………………… Date……………………..**

**Name of supervisor………………………………………...Date…………………….**

**Section A: Demographic Characteristics**

1. Sex (Tick) Male (1) Female (2)

2. Age in Years _____________________

3. Marital Status (Tick)

1. Single
2. Currently Married
3. Divorced
4. Widow/ widower

4. Religion (Tick)

1. Christian
2. Islam
3. Non-practicing
4. Others, specify __________________________

**Section B. Socio-economic characteristics**

5. Level of Education (Tick)

1. Never attended school
2. Did not complete primary school
3. Completed primary school but did not complete secondary school
4. Completed secondary school
5. Further studies after secondary school
6. Others, specify ___________________________

6. Main occupation (Tick)

1. Farmer
2. Small business (kiosk, kibanda)
3. Big business (shop)
4. Housewife
5. Salaried worker (teacher, police, chief)
6. Casual laborer
7. Others, specify ________________________

**Section C. Water sources in the household**

1. What is the main source of water used by members of your household?
2. Piped water to household
3. Public tap in the village
4. Borehole
5. Protected dug well
6. Unprotected dug well
7. Protected spring
8. Unprotected spring
9. Rainwater collection
10. Bottled water
11. Donkey pulled cart with small tank/drum
12. Tanker-truck
13. Surface water (river, dam, lake, pond, stream, canal, irrigation channels)
14. Other (specify)
15. How long does it take you to go to your main water source, get water and come back?
16. On premises
17. Less than 30 minutes
18. More than 30 minutes
19. Don’t know
20. Are you satisfied with the water supply?
21. Yes
22. No
23. I don’t know

**Type of sanitation facility in the household**

1. What kind of toilet facility do members of your household usually use?
2. Flush to piped sewer system
3. Flush to septic system
4. Pour-flush to pit
5. VIP/simple pit latrine with floor/slab;
6. Composting/dry latrine
7. Flush or pour-flush elsewhere
8. Pit latrine without floor/slab;
9. Service or bucket latrine
10. Hanging toilet/latrine
11. No facility, field, bush, plastic bag
12. Other (specify)
13. Do you share this facility with other households?
14. Yes
15. No
16. How many households use this facility?
17. Not shared (**1 HH**)
18. Shared family (**2 HH**)
19. Communal toilet (**3 HH or more**)
20. Public toilet (**in market or clinic etc**.)
21. Don’t know
22. Do you have children under three years old?
23. Yes
24. No
25. The last time (Name of the youngest child) passed stools, what was done to dispose of the stool?
26. Child used toilet/latrine
27. Put/rinsed into toilet or latrine
28. Put/rinsed into drain or ditch
29. Thrown into garbage
30. Buried
31. Left in the open
32. Other (specify)
33. I don’t know

**Observation based questions (To be done after the initial questions to ensure the flow of the interview is not broken)**

1. Please show me the containers you used yesterday for collecting water

List them

1. Please show me where you store your drinking water. **(A**re the drinking water containers covered or narrow necked?)
2. All are
3. Some are
4. None are
5. Please show me the toilet facility that is usually used by family members.
6. Toilet in use
7. Toilet not in use
8. Not observed
9. What is the floor of the toilet made of?
10. Block (stone)
11. Wood
12. Soil
13. Other (specify)
14. Are there faeces on the floor of the toilet?
15. Yes
16. No
